# Delivery of Multimodal Analgesia to Effectively Treat Acute Pain: A Review From Roma Pain Days

**DOI:** 10.7759/cureus.22465

**Published:** 2022-02-21

**Authors:** Magdi Hanna, Antonio Montero Matamala, Serge Perrot, Giustino Varrassi

**Affiliations:** 1 Department of Anesthesiology, University of London, London, GBR; 2 Department of Surgery, University of Lleida, Lleida, ESP; 3 Rheumatology, Cochin University Hospital, Paris, FRA; 4 Research, Paolo Procacci Foundation, Roma, ITA

**Keywords:** tramadol, dexketoprofen, drugs in fixed-dose combinations (fdc), multimodal analgesia, pain chronification, acute pain, low-back pain (lbp)

## Abstract

It is crucial that acute pain be promptly and adequately treated in order to prevent it from transitioning to chronic pain, a devastating and sometimes permanent condition that is challenging to treat and associated with disability, reduced quality of life, and depression. Guidelines for the treatment of acute low-back pain (LBP) are predicated on assumptions that all acute LBP is benign, temporary, and traditionally treated with a “wait and see” approach. LBP is far from a monolithic condition: etiology, the presence of underlying conditions, mental health status, social situation, patient’s age and occupation, and comorbidities all present different risk factors for chronic LBP that should be considered in treating acute LBP or other forms of acute pain. A multimodal approach to acute pain has been shown to be safe and effective. In particular, the combination product of oral dexketoprofen and tramadol has been shown effective in controlling acute pain, which spares the use of opioids and is well tolerated. Chronic pain must be viewed as a global health crisis, and the timely and adequate control of acute painful conditions is a good strategy to reduce its prevalence. Experts at Roma Pain Days discussed this important topic which is the foundation of this review.

## Introduction and background

Acute pain remains poorly managed, and its prevalence is underestimated. The inadequate management of acute pain is not acknowledged by most healthcare systems as being a particular problem. In many cases, acute pain is treated with the most familiar treatments rather than the most effective treatments. In a systematic review of 40 studies (n=5,116), nonsteroidal anti-inflammatory drugs (NSAIDs) were compared to codeine for managing acute postoperative pain. Adult outpatients had improved pain scores, better global assessments, and fewer adverse events with NSAIDs compared to codeine for acute pain management [1], yet codeine is often chosen for the treatment of acute painful conditions. The clinical assessment of acute pain in patients is not always reliable. A systematic analysis of 80 studies (n=20,496) in which pain assessments by clinicians were compared to patients’ self-reports of pain (within 24 hours of each other) found that 78% of studies underestimated patients’ pain, 1% overestimated it, and 21% matched the patient’s self-assessment [2]. A subset of those studies, limited to studies with >200 patients and using a randomized and/or blinded design, found that 91% of the studies underestimated patient pain, and the degree of underestimating pain increased with the severity of pain [2]. This suggests that the underestimation of pain, particularly severe pain, by healthcare professionals contributes to the undertreatment of pain. The aim of this review is to highlight some of the topics brought up by international pain specialists at Roma Pain Days in 2021.

Despite our growing understanding of pain mechanisms, neuroplasticity, and pharmacology [3], acute pain is still inadequately managed. The most frequently used drugs for acute pain control remain codeine and paracetamol (acetaminophen), although these are not necessarily the most effective or safest pain relievers [1]. Despite a wealth of studies of acute and chronic pain, scientific data are not reliably translated into clinical practice. While the armamentarium for pain control is large, there are few new compounds and no optimal agents. A growing awareness of multimodal pain control regimens has emerged, which allows clinicians to treat multiple pain mechanisms with two or more agents with complementary mechanisms of action. Clinicians must understand and deploy these multimodal regimens better, even for acute painful conditions [4]. In finding solutions to treat acute pain better, it is imperative to know drugs better but, perhaps even more important, to know patients better.

## Review

Low-back pain (LBP) is prevalent, often inadequately treated, is a major cause of disability, and has been recognized as a global health issue [5]. LBP is not restricted to higher or lower-income countries but occurs all over the world in all populations. It can affect men and women, old and young, fit and frail. Pain is the main factor of disability associated with LBP [6]. While LBP can become chronic, it may also follow a cycle of resolution and recurrence, which should also be considered a form of chronic LBP. About three-quarters of all LBP patients experience frequent pain recurrences [7]. Even more alarming is the fact that about one-third of LBP patients experience levels of pain intensity described as severe to unbearable [7]. Complicating LBP treatment is the fact that comorbidities are common with LBP, and LBP may occur as a primary condition or secondary to trauma, cancer, or other conditions. Patients diagnosed with LBP may stay home from work, retreat from ordinary daily activities, and perhaps even experience temporary disability, all of which promote a lack of exercise and social isolation. In the home setting, consideration and empathy for a person with LBP tend to be short-lived, leading to conflicts. These conditions do not facilitate recovery; on the contrary, they can exacerbate the LBP and cause discouragement or depression in the patient [8]. The ramifications of LBP go far beyond isolated individual cases. The enormous costs of LBP to the global economy are hard to measure and must include both healthcare expenditures and lost productivity [6].

There are over a dozen national and international guidelines for managing acute LBP [9]. The general objective behind these guidelines, particularly the older ones, is the functional recovery of the patient back to previous levels of activity. Guidelines also stress the importance of preventing acute LBP from developing into chronic LBP, which is more challenging to treat. In the United States, LBP treatment is predicated on the notion of risk avoidance in that opioid use is discouraged [10]. While the United States has a unique approach to opioid analgesia, solutions to LBP in all parts of the world tend to favor simple, straightforward, and inexpensive care. However, such care is not always effective and rarely produces long-lasting results. Guidelines may also differ. As an example, the use of paracetamol to treat LBP is recommended in some guidelines, while others find it ineffective. Most guidelines approach LBP with an incremental treatment strategy; that is, care is administered one step at a time, advancing only when the previous step fails [11]. The step-by-step method may force a patient to go through a series of ineffectual and time-consuming steps (reassurance without treatment, waiting period, telephone consultation, and so on) before the patient receives what might be considered more meaningful medical treatment. This incremental approach denies a patient in moderate to severe pain adequate treatment until the requisite initial steps fail, which means an unnecessary delay in treatment for some patients.

From our vantage point today, it is time to challenge the assumptions on which these guidelines are based (see Table 1). The first assumption of most LBP guidance is that acute LBP is a benign condition that resolves in a matter of days without much fuss, even if no treatment at all is provided. Many guidelines are based on the erroneous notion that all acute LBP is more or less the same condition and that patients are all at approximately equal risk to progress to chronic LBP. Most guidelines work on the assumption that acute LBP will resolve in a few days, so a conservative approach is the soundest. The step-by-step approach is based on the idea that passive observation is preferable to more rigorous assessment and more rapid treatment [12]. It may be argued that guidelines have been developed from fundamental assumptions, selectively using scientific data to support them rather than using scientific data to formulate the fundamental principles. Perhaps the best evidence that these guidelines have failed is that chronic LBP has increased exponentially, namely by 10% each year, over the past 10 years [12].

**Table 1:** Current guidelines of low-back pain appear to be based on erroneous fundamental assumptions encountered in clinical practice.

Assumption	Challenge	Comments
All acute LBP is benign	Acute LBP can transition into chronic LBP	Acute LBP can be complex and individual patient factors that could contribute to LBP must be considered in the treatment
Acute LBP is temporary	Not all acute LBP will resolve on its own	Acute LBP can transition into subacute and chronic LBP and lack of prompt adequate treatment may facilitate rather than prevent this
Acute LBP is short-lived	LBP may last for weeks (subacute)	When LBP becomes subacute, there is a high risk of chronification
Wait and see is a good approach	Patients may be in pain, and they can be at risk for chronic LBP	Risk factors for chronic LBP include pain intensity, pain duration, previous experiences, and psychological factors
Step-by-step is conservative and cost-effective	It is not expensive in the short run but it exposes acute LBP patients to the risk of pain becoming chronic—and that can be very expensive	If acute LBP becomes chronic, the costs are passed on to the other treatment providers and society at large (lost productivity)

The increased and increasing prevalence of chronic LBP suggests core problems in the guidelines. It is the opinion of the authors that the guidelines fail to recognize the complexity of acute LBP and assume that most acute LBP patients will recover with little or no treatment. While recovery from acute LBP is certainly possible, it should not be presumed. Not all patients with acute LBP will follow the same timetable or trajectory. It must be recognized early in care that certain acute LBP patients have specific risk factors for the development of chronic pain. Thus, what is urgently needed is a more patient-centric approach to acute LBP. Patient-centric care emphasizes that patient factors contribute to the totality of the patient’s pain experiences and must be considered when determining effective analgesic treatment. When treating acute LBP, clinicians should assess pain intensity, consider the duration of the painful symptoms, take a patient history with special emphasis on previous LBP experiences, examine the patient physically, and evaluate psychological factors that could contribute to pain [12].

**Figure 1:**
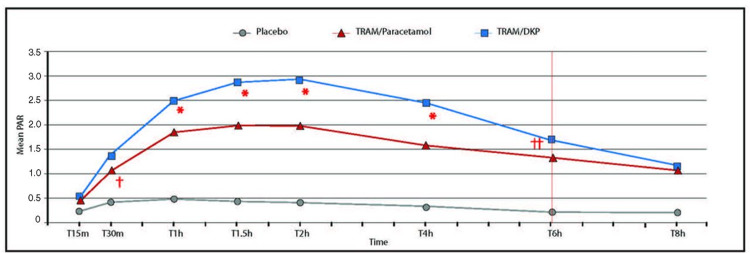
Eight-hour pain relief using placebo (gray), tramadol-paracetamol (acetaminophen) (red), and tramadol-dexketoprofen (blue). Treatment with fixed-dose combination of tramadol-dexketoprofen (75 mg-25 mg) was significantly better. Red asterisk: time assessment occurred; Single red cross: statistically significant TRAM/paracetamol vs. TRAM/DKP (p<0.0006); Double red cross: statistically significant TRAM/paracetamol vs. TRAM/DKP (p<0.00086) Abbreviations: m, minutes; h, hours; PAR, pain relief; TRAM, tramadol; DKP, dexketoprofen. This figure is from Gay-Escoda et al., 2019 [13], used here in accordance with the Creative Commons Attribution-Noncommercial (CC BY-NC 4.0) license.

The deliver multimodal analgesia to effectively treat acute low-back pain (DANTE) study is a follow-up study to the tramadol hydrochloride and dexketoprofen trometamol for the oral treatment of moderate to severe acute pain following removal of impacted lower third molar (DAVID) study, both of which compared an analgesic regimen of tramadol/dexketoprofen 75/25 mg versus tramadol/paracetamol 75/650 mg in a placebo- and active-controlled single-dose clinical study, which enrolled 653 adults with moderate to severe pain associated with oral surgery. The primary endpoint was the total pain relief (TOTPAR) at six hours; the response was defined as pain reduction >50% [13]. Tramadol/dexketoprofen at both doses was effective and superior to both tramadol/paracetamol and placebo in relieving dental pain; tramadol/dexketoprofen had a more rapid onset of action and provided more durable analgesia (Figure 1) [13].

Multimodal therapy has been defined by the International Association for the Study of Pain (IASP) as the concurrent use of separate, therapeutic interventions with different mechanisms of action within one discipline aimed at different pain mechanisms [14]. The idea behind multimodal analgesia is the ability to target two or more points in the nervous system simultaneously. It is misleading to think of multimodal analgesia as merely the concurrent use of two or more agents. For example, codeine and paracetamol used together cannot be considered multimodal treatment because this treatment regimen fails to target two different pain mechanisms. Multimodal regimens typically use agents with complementary mechanisms of action that exert a synergistic benefit [15]. Furthermore, multimodal analgesia has the potential to improve outcomes, reduce adverse events, and provide high-quality pain control.

Multimodal analgesia has become the standard of care for total joint arthroplasty and produces adequate analgesia with fewer side effects than opioid monotherapy. For total joint arthroplasty, the main types of analgesics used in combination include NSAIDs, paracetamol, corticosteroids, gabapentinoids, local anesthetics via local infiltration, and peripheral nerve blocks [16]. However, the paradigm of multimodal pharmacologic regimens is not limited to analgesia and has been used in oncology, respiratory diseases, and other conditions. A meta-analysis of various combination therapies for cardiovascular disease prevention (antihypertensives, statins, aspirin) found the combination approach substantially reduced cardiovascular disease, myocardial infarction, revascularization, stroke, and cardiovascular death even after adjusting for metabolic risk factors [17].

Tramadol is a weak µ-receptor agonist with a metabolite, o-desmethyltramadol, that has a 200-fold greater µ-receptor affinity [18]. Tramadol is also a serotonin and norepinephrine reuptake inhibitor [19]. Dexketoprofen is a highly effective cyclo-oxygenase (COX)-1 and COX-2 inhibitor and thus inhibits prostaglandin production.

Numerous clinical studies support the use of tramadol/dexketoprofen as a safe and effective analgesic [13,20-22]. In a single-center observational study, tramadol/dexketoprofen 75/25 mg was compared head-to-head to diclofenac/thiocolchicoside 75/4 mg for moderate to severe acute LBP [23]. Tramadol/dexketoprofen provided significantly greater and longer-duration analgesia at day three (93.2% vs. 73.7%) and day seven and had more responders at all time points in the study. Reductions in neuropathic pain as measured on the Douleur Neuropathique 4 (DN4) survey were significantly greater with tramadol/dexketoprofen as well (-62.7 vs. -39.7, p<0.0001) [23].

At the time of this presentation, recruitment is ongoing in the DANTE study, which has 312 randomized acute LBP patients. The recruitment goal is to screen a total of at least 612 patients in order to enroll 510. There are currently 54 international sites participating in the trial, located in Croatia, Estonia, Hungary, Latvia, Poland, and Spain. As of September 2021, 291 patients have already completed the study. The target for enrollment completion is in the second quarter of 2022.

Chronic pain must be considered a global public health crisis since it results in more disability than cancer, heart disease, AIDS, stroke, and chronic obstructive airway disease combined [24]. Yet chronic pain has become so ubiquitous in our society that it is easy to minimize how devastating a condition it is. Chronic pain in certain situations may be preventable, and it is imperative that clinicians treat acute pain with that objective in mind. In this context, it is important to recognize that the definition of chronic pain is not just pain that persists for more than three months, but it is also pain that recurs within three or more months [25]. Many people with acute LBP develop a form of recurrent LBP, which must be considered a type of chronic LBP. Thus, clinicians have only a relatively narrow window for targeting acute pain in order to prevent chronic pain from developing.

Clinicians may consider using multimodal approaches to acute pain syndromes, particularly the prevalent condition of acute LBP that may develop into subacute and chronic forms of LBP, which are much more challenging to treat and may be a source of disability.

## Conclusions

The optimal approach for acute LBP must avoid the “wait and see” approach favored in some guidelines. Passively monitoring pain with the expectation that LBP will resolve on its own can set the stage for chronic LBP. More immediate, decisive, and patient-centric actions are required, particularly for pain control. A patient-centric approach recognizes the individual risk factors for chronic LBP. Acute LBP can occur in anyone, but patients with certain underlying conditions, comorbidities, mental health issues, age, frailty, occupations, and socioeconomic situations may be at an elevated risk for a transition from acute to chronic LBP. Even for acute LBP, multimodal analgesic regimens should be preferred over monotherapies. Many multimodal approaches, such as tramadol/dexketoprofen, have been shown to be effective and should be used earlier rather than later in the course of acute LBP care. The overarching goal in treating acute LBP is providing immediate pain relief while reducing the likelihood that chronic LBP develops. Chronic pain is debilitating, associated with dysfunction, and can be challenging to treat. Prompt, safe, and effective treatment of acute pain may reduce the prevalence of chronic pain. Further studies are warranted.
